# Correlated Mutations: A Hallmark of Phenotypic Amino Acid Substitutions

**DOI:** 10.1371/journal.pcbi.1000923

**Published:** 2010-09-16

**Authors:** Andreas Kowarsch, Angelika Fuchs, Dmitrij Frishman, Philipp Pagel

**Affiliations:** 1 Lehrstuhl für Genomorientierte Bioinformatik, Technische Universität München, Wissenschaftszentrum Weihenstephan, Freising, Germany; 2 Institut für Bioinformatik und Systembiologie/MIPS, Helmholtz Zentrum München – Deutsches Forschungszentrum für Gesundheit und Umwelt, Neuherberg, Germany; Max-Planck-Institut für Informatik, Germany

## Abstract

Point mutations resulting in the substitution of a single amino acid can cause severe functional consequences, but can also be completely harmless. Understanding what determines the phenotypical impact is important both for planning targeted mutation experiments in the laboratory and for analyzing naturally occurring mutations found in patients. Common wisdom suggests using the extent of evolutionary conservation of a residue or a sequence motif as an indicator of its functional importance and thus vulnerability in case of mutation. In this work, we put forward the hypothesis that in addition to conservation, co-evolution of residues in a protein influences the likelihood of a residue to be functionally important and thus associated with disease. While the basic idea of a relation between co-evolution and functional sites has been explored before, we have conducted the first systematic and comprehensive analysis of point mutations causing disease in humans with respect to correlated mutations. We included 14,211 distinct positions with known disease-causing point mutations in 1,153 human proteins in our analysis. Our data show that (1) correlated positions are significantly more likely to be disease-associated than expected by chance, and that (2) this signal cannot be explained by conservation patterns of individual sequence positions. Although correlated residues have primarily been used to predict contact sites, our data are in agreement with previous observations that (3) many such correlations do not relate to physical contacts between amino acid residues. Access to our analysis results are provided at http://webclu.bio.wzw.tum.de/~pagel/supplements/correlated-positions/.

## Author Summary

Point mutations (i.e., changes of a single sequence element) can have a severe impact on protein function. Many diseases are caused by such minute defects. On the other hand, the majority of such mutations does not lead to noticeable effects. Although previous research has revealed important aspects that influence or predict the chance of a mutation to cause disease, much remains to be learned before we fully understand this complex problem. In our work, we use the observation that sometimes certain positions in a protein mutate in an apparently correlated fashion and analyze this correlation with respect to mutation vulnerability. Our results show that positions exhibiting evolutionary correlation are significantly more likely to be vulnerable to mutation than average positions. On one hand, our data further support the concept of correlated positions to not only be associated with protein contacts but also functional sites and/or disease positions (as introduced by others). On the other hand, this could be useful to further improve the understanding and prediction of the consequences of mutations. Our work is the first to attempt a large-scale quantitation of this relationship.

## Introduction

Most of the missense mutations do not lead to an appreciable phenotype when they occur in nature or are introduced experimentally. There are, however, numerous counterexamples where even a subtle change of the primary protein sequence results in severe phenotypical effects – i.e. genetic disease. Understanding the underlying mechanisms which determine the link between genotype and phenotype is the key issue in developing strategies for diagnosis and treatment of hereditary diseases.

Databases such as OMIM (Online Mendelian Inheritance in Man) or HGMD (Human Gene Mutation Database) provide a wealth of information [1], [2] about phenotypes associated with thousands of known human mutations. These databases assist researchers in analyzing the molecular basis of human disease. With the current quest for the “1000 Dollar Genome”, there is no doubt that entire patient genomes will be available in the near future which will substantially accelerate the discovery of new mutations of unknown significance. In such a situation the question “What does this mutation mean for a patient's health?” will become more and more practical for the affected individuals and physicians.

### Properties of “disease proteins”

What rules determine the spectrum of allowed mutations, and why do mutations cause disease in some genes while other genes appear to be more tolerant to substitutions? Much effort has been invested in answering such questions and promoting our understanding of the underlying mechanisms which rule the complex network of factors contributing to human disease. In particular, we would like to understand what properties or features are shared by disease-associated genes.

In addition to numerous careful experiments on individual genes and proteins, with the advent of high-throughput technologies such as genomics, proteomics and, more recently, metabolomics large bodies of experimental data have been analyzed towards this end. It has been shown that genes and proteins, which are known to be involved in a large variety of diseases and syndromes, differ from genes without such association in many aspects. Disease genes have a broader phylogenetic distribution, tend to be longer on average, and more of them have homologs in other mammals compared to the average human gene [3]. Disease-related proteins have been found to be better conserved and their synonymous substitution rates are significantly higher than expected [4], [5]. Further contributions demonstrated that disease proteins have less designable folds, tend to have isoelectric points closer to neutrality, contain more alternating hydrophilic/hydrophobic stretches compared to the average human protein and have a higher tendency to aggregate [6]. “Disease genes” are highly expressed in a small number of tissues, and their encoded proteins are more likely to be secreted and mutated in genetic diseases with Mendelian inheritance [7]. Finally, genes associated with inherited disease mutations are less likely to be essential and display an intermediate level of connectivity on protein interaction networks [8].

### Identification of critical residues

Knowing what genes and proteins are involved in disease is only one part of the challenge. Clearly, not every site of a protein is equally vulnerable when hit by mutations. While some parts of the molecule will remain functional even after substantial changes of the primary sequence, other positions cannot be changed at all without serious consequences. Evolutionary conserved and functionally important residues, such as those in active centers of enzymes, as well as residues important for preservation of the protein's overall stability, in particular those located in buried positions, have been shown to be frequent targets of disease-associated mutations [9], [10], and multiple prediction techniques based on both structural and sequence features of proteins have been suggested to distinguish benign mutations from those implicated in inherited disease. Notable tools to combine multiple lines of evidence to produce more reliable prediction include SIFT and PolyPhen [11], [12] (see [13] for an excellent review).

### Correlated mutations

The phenomenon of correlated mutational behavior between columns of a multiple sequence alignment has been described for many years for both DNA/RNA and protein sequences [14]–[16]. For proteins, the initially hypothesized notion of the underlying biological event was, that an unfavorable amino acid change in a structural contact site may go without negative consequences if its direct binding partner is simultaneously mutated in such a way that the original interaction is salvaged (compensatory mutation).

Accordingly, the analysis of such correlated mutations has been traditionally employed for the identification of residue contact pairs within or between different protein chains. The first approach to detect co-evolving residues in a multiple sequence alignment was proposed in 1994 [17]. Many other methods have been reported since then and evaluated with respect to their potential of predicting residue-residue contacts [18]–[24]. However, despite significant progress in method development, comparative studies have shown that prediction accuracies for structural contacts hardly exceed 20% with any of these methods [25], strongly limiting the application of the predicted contacts as structural constraints in *ab initio* structure prediction.

While some authors have explained the low contact prediction accuracies with the difficulty of differentiating correlation signal from random noise [26], [27], recent studies indicate that co-evolution of amino acids in fact may originate not only from structural contacts but from a much broader range of biological reasons. Using Statistical Coupling Analysis Ranganathan et al. (2005) detected correlation rules in the WW domain which describe aspects of the fold architecture going beyond simple protein contacts. They introduced the concept of a correlation backbone in the fold which they claimed was nearly sufficient to describe the architecture without additional information [28]. They impressively demonstrated the power of this idea by synthesizing artificial WW domains solely based on the previously derived correlation model and showing that a substantial percentage of these designed polypeptides were able to fold into functional WW domains *in vitro* [29].

In addition, further contributions have demonstrated that correlated mutations may also occur due to reasons related to protein function. Gloor et al. analyzed 12 mutations of the ATP synthase 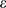 subunit and 7 mis-sense mutations of the homeodomain and came to the conclusion that certain co-evolving residues are more likely to be functional sites and thus possibly more likely to be related to disease [18]. Within a study on the Hsp70-Hop-Hsp90 system, regions previously known to be functionally important could be identified based on residue co-evolution [30]. Additionally, the authors pointed out that co-evolving amino acids were often found to be in close proximity to functionally important sites. Similar results were obtained in an analysis of correlated mutations within the cytochrome c oxidase subunit I where many co-evolving residues were found adjacent to hypothesized proton pumping channels [31]. In a recent publication Lee et al. provided further evidence for the hypothesis that correlated mutation may be related to functional importance in an analysis of 44 selected protein families [32].

All together, these results indicate that co-evolving residues may be both structurally or functionally important positions within protein folds and therefore could be likely targets for disease-associated point mutations. Here, we present the first comprehensive analysis of human disease mutations with respect to co-evolving residues using all known point mutations and proteins currently available in the Human Gene Mutation Database (HGMD). Our data confirm that correlated mutations go well beyond contact prediction and are a hallmark of amino acid positions leading to disease when affected by mutation.

## Results

For our analysis, we used all human proteins known to be affected by at least one disease causing point mutation according to HGMD annotation and for which at least 30 orthologous proteins of sufficient sequence diversity were available for building a multiple sequence alignment. 1153 proteins fulfilled all requirements and were analyzed for correlated mutations using the OMES algorithm. In addition, we repeated all analyses on a more rigorous dataset using a cutoff of 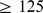 proteins per ortholog cluster which left us with 855 human disease proteins. Using these two data sets, we identified 62 365 and 46 022 residues as correlated with other positions, respectively. A total of 14211 and 10508 positions were found to be disease-related in these two sets.

### Co-evolving positions are enriched in disease mutations

As stated above, our work is motivated by the observation that co-mutation of residues over the course of evolution may not to be restricted to protein contacts but rather be the result of other types of functional association among residues. Accordingly, our first goal was to test the hypothesis that point mutations affecting correlated residues are more likely to result in disease than expected by chance (i.e. compared to random positions).

Using all residues represented in the datasets described above, we produced contingency tables of correlatedness vs. known disease-mutations. Based on these tables, we computed the background rates of disease mutations to be 0.019% for random positions and 0.032% for correlated positions (0.0195 and 0.0325 for the clusters 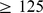). In other words, correlated residues were found to be 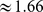 times more likely to be known disease positions than expected by chance translating to a log odds value (LOD, 
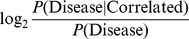
) of 0.73.

For the clusters 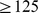, the relative increase was found to be 1.66 (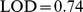). Fisher's exact test for count data confirmed that the observed difference is highly significant in both cases (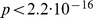; see Table 1 for summary). As the stringent data did not yield a substantial gain over the less strict set, we are reporting the results of the latter (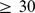) data in the subsequent text. All results for the stringent set are reported in the Text S1.

**Table 1:** Influence of the alignment threshold on disease enrichment in correlated positions.

Alignment cutoff	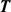	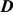	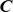	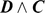	LOD	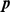 -value
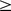 30	741436	14211	62365	1988	0.73	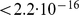
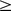 125	538283	10508	46022	1498	0.735	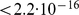

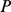
-values were computed using Fisher's exact test. As both selection methods achieve positive LOD score and highly significant 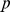-values this result indicates that disease-causing mutations are overrepresented in correlated positions. 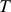: number of all residues; 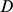: number of disease mutations; 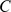: number of correlated positions; 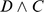: number of correlated residues affected by disease-associated mutations.

**Figure 1:**
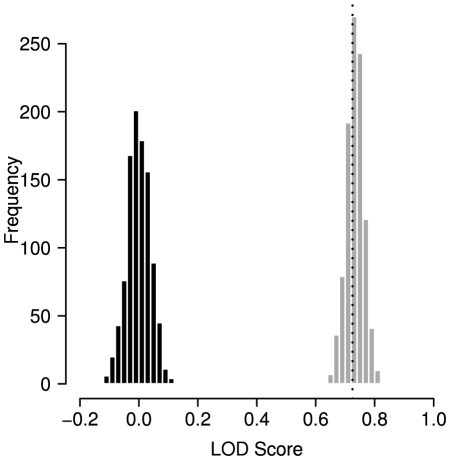
Disease mutations occur significantly more often in correlated positions than expected. Black: Empirical background distribution obtained by 1000 permutations (random expectation). Grey: Bootstrap distribution of observed LOD. Dotted vertical line indicates the observed LOD obtained by an alignment cutoff of 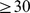.

Figure 1 shows the empirical background distribution of LOD values generated by 1000-fold permutation of correlation scores in comparison to the observed LOD. In addition, we show the bootstrap distribution of the observed LOD generated by 1000fold resampling of individual positions from all multiple sequence alignments (same as 8.1 in Text S1). Clearly, the observed value is far outside the background distribution.

In order to cross-check our findings, we also computed the LOD for a set of positions that are highly *unlikely* to be associated with disease because of evolutionary accepted mutations (see Materials and Methods). In this data we find an LOD of −1.26 indicating that these positions are clearly *under*represented in the set of correlated positions.

### Impact on individual proteins

After having shown that correlated positions are in general significantly more likely to be hit by disease-causing point mutations we sought to investigate the implications of this finding for individual proteins. We repeated the above analysis for all 1153 proteins separately. As both the number of known disease-mutations and the degree of correlation varies among proteins, one would expect that for some proteins, correlation is strongly associated with disease-susceptibility while in others no such signal can be detected. In fact, analysis of individual proteins also yields an arithmetic annoyance: proteins with a very low number of known disease mutations have a very large chance that none of them is located in a correlated position simply because of the small sample size, resulting in an 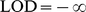. These cases were excluded from the following analysis as no valid statistical analyses could be carried out. In total, an LOD score could be calculated for 524 proteins of the data set and 629 proteins obtained no score. The analysis of the proteins for which our approach failed shows that 50% (315) of these proteins have only one known disease mutation in HGMD and for only 3.8% (24) more than 9 disease-related substitutions were available.

**Figure 2:**
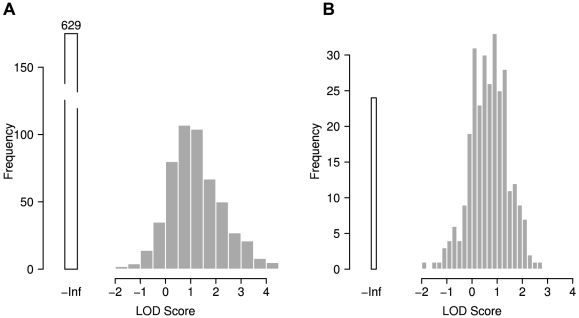
Disease mutations are overrepresented in correlated positions. Distribution of log odds (LOD) scores for individual proteins. All proteins for which no score could be obtained were excluded. A: All proteins; B: proteins with 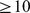 disease mutations. The bars at -Inf represent cases where no position was both correlated and associated with disease, resulting in an LOD of 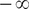, as discussed in the main text.

The LOD distribution for individual proteins is depicted in Figure 2 (Alignment threshold 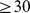; see Text S1 for threshold 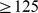). Only a small fraction of proteins (10% in Figure 2a) in our data set had LOD values 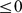 and a clear majority of proteins had at least slightly positive LODs. In some cases, we observed LOD scores 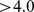 which represents an increase of 1500% over expectation. Taken together, these numbers indicate that, except for cases with very few known disease mutations, the global result applies to the majority of individual proteins.

### What about conservation?

Detection of correlated residue pairs is not entirely independent of the degree of conservation of the respective positions. Depending on the algorithm used, substantial crosstalk between conservation and correlation can be observed [25]. Although the OMES method is reputed to be among the more robust approaches with respect to interactions with conservation, we investigated the degree to which sequence conservation affects our results. This is especially important as evolutionary conservation of a sequence region is generally taken as an indication of functional importance and thus would represent a bias in favor of the hypothesis under test.

We calculated two different measures of conservation for each position in the multiple sequence alignments. The first method (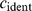) computes fractional identity to the human (reference) residues in each column of the MSA. The second procedure (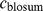) computes a conservation score based on the BLOSUM62 amino acid substitution-matrix [33] for each column.

**Figure 3:**
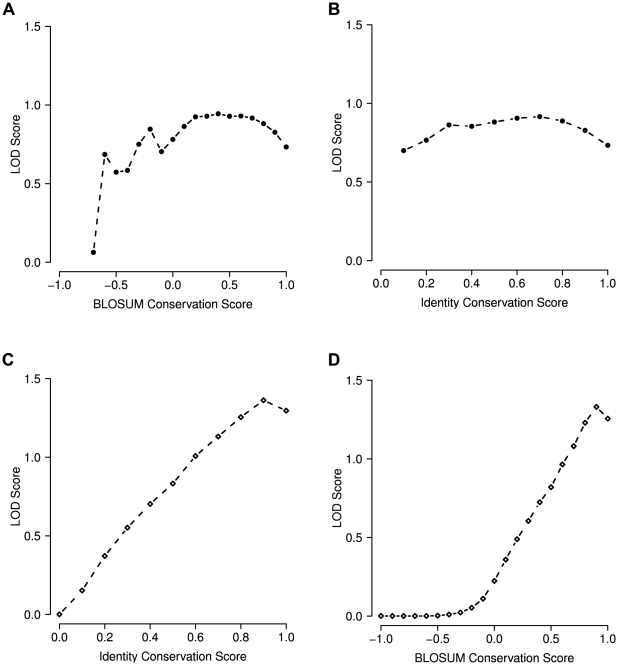
Interdependence between correlation and sequence conservation. LOD distribution for different conservation thresholds: (A, B) LOD for correlated residues at different levels of conservation. (A) BLOSUM conservation score, (B) fractional identity. Each dot represents the LOD score achieved using a specific conservation cutoff. A cutoff of 0.4 indicates that for the calculation of the global LOD score only the residues which have a conservation score 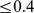 were taken into account. (C, D) LOD scores for sequence conservation *irrespective* of residue correlation. Here, a cutoff of 0.4 indicates that the global LOD represents all positions with a conservation score 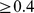. The LOD remains largely stable over wide ranges of sequence conservation (A,B). Residue conservation yields LODs similar to correlation for intermediate levels of conservation and performs better for very high conservation.

To evaluate the interaction between correlation and conservation we applied stepwise filtering on conservation. Starting from the full dataset, we decremented the conservation threshold 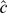 in small steps thus removing more and more of the top conserved columns from the MSA. The background frequency 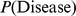 as well as the observed frequency 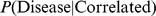 of all positions was re-computed for all of these filtered datasets and the corresponding LOD values were computed. Figure 3(A,B) shows LOD values plotted against the respective conservation cutoff 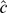. E.g. 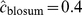 indicates that for the calculation of the global LOD score only residues with 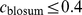 were taken into account.

For comparison, we also computed the LOD score of *conservation* with respect to disease mutations. Our initial intuition was that conservation would probably be a much more potent indicator of functional importance than correlation and thus yield substantially higher LOD values for being affected by disease mutations. We performed this analysis at different conservation cutoffs to get an impression of the degree of conservation required to detect functional importance.

As expected, we found well conserved positions to be clearly enriched in disease mutations, and we observed a correlation between the degree of conservation and the LOD score (Figure 3C,D). Depending on the conservation measure and cutoff, well conserved positions were 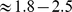 times as likely to be affected by known disease mutations as random positions (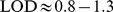). As expected, conservation clearly outperforms correlation. Given the obvious link between conservation and functional importance, the numbers for correlated positions are surprisingly high. Furthermore, the LOD values for correlation remain remarkably stable over a fairly wide range of conservation thresholds indicating that the correlation signal is not merely an artifact caused by relatively well conserved positions which happen to also correlate. Taken together, these results suggest that evolutionary conservation is a useful measure for the assessment of disease-susceptibility and thus functional significance of amino acid positions in a protein.

### Non-contact correlations?

Other groups have previously demonstrated that correlated positions without physical contact do occur in protein structures [28], [34] and, in a recent study, Noivirt-Brik *et al.* have demonstrated the emergence of long-range interactions in lattice models of proteins [35].

In the dataset analyzed in our own work, we found that only 2714 out of 16555 (16.4%) of correlated pairs had a distance of less than 5.5Å which would imply physical contact. If the distance threshold is relaxed to a generous 8.0Å, still only 17.4% are in proximity. Thus, even applying a very permissive threshold, the *majority* of correlations is observed between residues which are not in direct contact – an observation compatible with the hypothesis of functional correlations.

Of course, many positions correlate with more than one other residue and accordingly, some of these correlations coincide with contacts while others do not. In our data, 29.6% of all correlated positions had at least one contact correlation. For the positions which were found to be both correlated and relevant for disease 31% had at least one contact 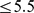
Å.

Finally, we wanted to determine if non-contact correlations are enriched in disease mutations. Out of 4960 correlated residues without a single contact, 252 (5.0%) were also disease positions. This corresponds to 1.3 fold increase over the expectation (LOD  =  0.39). Due to the small sample size the latter is not statistically significant. Nevertheless, the trend is encouraging and points in the direction of the hypothesis of functionally relevant non-contact correlations. Table 2 summarizes the results for correlation, conservation and residue contacts.

**Table 2:** Summary of performance.

subset	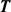	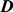	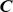	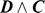	LOD	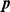 -value
Conservation ( 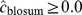 )	741436	14211	459030	10273	0.22	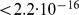
Conservation ( 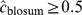 )	741436	14211	144591	4892	0.82	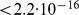
Conservation ( 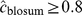 )	741436	14211	53443	2402	1.23	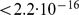
Contact	70589	2747	59079	2522	0.13	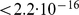
Correlation	741436	14211	62365	1988	0.73	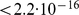
Correlation ( 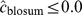 )	287029	3961	16271	388	0.78	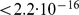
Correlation ( 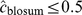 )	599322	9328	49254	1463	0.93	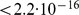
Correlation ( 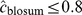 )	688790	11799	61802	1955	0.88	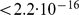
Correlation, non-contact	70589	2747	4960	252	0.38	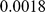

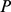
-values were computed using Fisher's exact test. 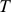: number of all residues; 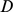: number of disease mutations; 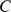: number of correlated positions; 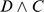: number of correlated residues affected by disease-associated mutations.

### Comparison with active sites

The most plausible explanation for a connection between correlated mutations and disease mutations is that correlated mutations indicate functional relevance of the respective residues. So on one hand, many of the disease positions are probably functionally active themselves or correlate with functionally important positions in the protein. That would imply that we should find significant enrichment of active sites in correlated position, too.

We used the SwissProt feature annotation to test this hypothesis and found that 3.7% of functional sites are annotated with at least one disease associated point-mutation in HGMD. That means that functional sites are roughly twice as likely to host disease mutations than expected. We carried out an analysis of enrichment in correlated positions with the functional site data and found it even stronger than for the disease mutation data (LOD  =  1.04, 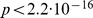). When active sites and HGMD mutations are combined into a single set the resulting enrichment lies between the results for disease and functional sites (LOD  =  0.88, 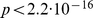). So both types of information show the same trend to lie in correlated positions.

Next, we asked the question if a position would be more likely to be involved in disease if it was correlated with another disease site or a known functional residue. We found that residues which are correlated with a disease position or a functional site are 5.2 and 2.9 times more likely to be disease positions themselves than randomly chosen residues, respectively. These numbers indicate that not only is correlated mutation itself an indicator of disease-relevant sites but apparently functional/disease positions seem to be preferentially correlated with each other. As for the disease positions analyzed above, we found that only a modest fraction (18%; 5.5 Å threshold) of functional positions which show significant correlation are involved in physical contact and thus the majority are non-contact correlations.

### Structural preferences

So far, correlation was the only variable taken into account in our analysis, but other structural features may also contribute to the potential of a correlated residue to be associated with disease. In order to test if basic structural features of a position affect our results, we investigated the accessibility of a residue in this context. This analysis was carried out in the subset of proteins with known structure which is much smaller than the full data set. The global LOD for disease in correlated positions in this smaller subset is 0.39. Interestingly, correlated residues that were exposed showed an LOD of 0.73 while partially and fully buried correlated residues had LOD of only 0.42 and 0.1, respectively.

Another obvious structural condition is the local secondary structure. Our analysis revealed that correlated positions are most likely to be associated with disease when they are embedded in an 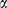-helix (LOD = 0.47) followed by turns (LOD = 0.37) and much weaker in in 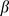-sheets (LOD = 0.09). This ranking is in part connected to the different accessibility found in these secondary structures: helices and turns are known to exhibit a much larger fraction of exposed residues than beta sheets and our sample is in accordance with this. Furthermore, in our data, the probability of a position being associated with disease is significantly negatively correlated with accessibility and the same is true for the probability of showing co-evolution with another residue. In a logistic regression model of the disease probability vs. accessibility and secondary structure, the secondary structure just barely achieved significance (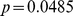) while accessibility was highly significant (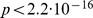). For the probability of a residue to co-evolve with another one, secondary structure was not a significant predictor (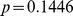) while accessibility was highly significant again (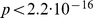).

As we have seen above, the enrichment of correlated disease residues is more pronounced in exposed sites. This observation appears plausible because much of the functional features of a protein are located on its surface: regulatory modifications, interactions with other proteins and binding sites for substrates need to be accessible. As we also find co-evolving residues to favor accessible locations, the above numbers come as no surprise.

### Contributing to prediction

**Figure 4:**
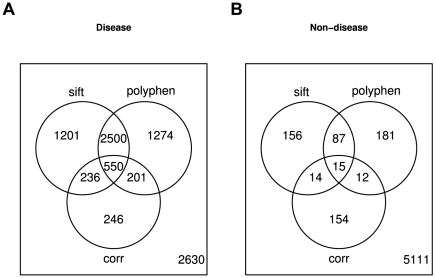
Venn diagrams of SIFT, PolyPhen and correlated mutations in (A) disease and (B) non-disease positions. For SIFT and PolyPhen, maybe and possibly damaging were treated as non-damaging.

Phenotypic consequences of mutations are determined by many different factors like functional role of the protein, specific amino acids involved, structural properties etc. Accordingly, no single feature can be expected to be a strong predictor on its own and correlation is no exception. Tools like SIFT [36] or PolyPhen [12] combine different signals into an integrated prediction of phenotypic effects of mutations. Currently, correlated mutation is not among the features analyzed by these programs. Based on our results presented above, we would expect that adding the correlation signal to these tools would further improve their predictions. A full assessment of this expectation would require access to the source code of these tools, which we did not have. On the other hand, we can analyze the overlap between predictions by integrated tools and the correlation data in order to estimate if the correlation data is fully included in these predictions or not. We compared predictions by SIFT and PolyPhen to the correlation signal in a set of 813 proteins featuring 8838 known disease mutations and 5730 substitutions unlikely to cause disease (see Materials and Methods). While the majority of the correlated positions in the disease mutation set were predicted to be damaging by either or both of the prediction programs, roughly 20% of the correlated positions were not. These numbers suggest that the correlation data is at least partially complementary to the results produced by both tools and thus has the potential to improve predictions (Figure 4). Furthermore, we carried out a multiple logistic regression, modeling the probability of disease association by the predictions by SIFT, PolyPhen and correlation and found all three terms to make highly significant contributions to the model (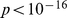).

### Case studies

In the previous sections we analyzed the dependence between co-evolving positions and known disease mutations. We were able to demonstrate that correlated mutations are associated with disease significantly more frequently than expected and found similar results for sites known to be functionally active. In the following case studies we present two examples of proteins with long-distance correlations and discuss the functional aspects of the residues involved.

### Proline dipeptidase

The human protein PEPD (UniProt: PEPD_HUMAN) is a proline dipeptidase which plays an important role in the recycling of proline during the final stages of degradation of collagen and dietary proteins. The enzyme hydrolyzes dipeptides with a prolyl or hydroxyprolyl residue in the C-terminal position. For catalytic activity, binding of 2 manganese ions per subunit is required as a co-factor [37], [38]. Swissprot annotation marks residues D276, D287, H370, E412 and E452 as responsible for manganese binding [39], [40].

Mutations of the PEPD protein have been identified as the cause of autosomal recessive prolidase deficiency (PD). E.g. Ledoux et al. have characterized several disease-causing point mutations in the PEPD protein [41]. Their data show the different extent of enzyme inhibition by these mutations. For instance, the R184Q mutations resulted in a residual activity of 7.4% compared to the wild type enzyme. The G278D and G448R mutations caused complete abrogation of peptidase activity. Other sources have found residues D276, S202 and E412 to cause the same disorder when hit by point mutations [42]–[44].

The phenotypic consequences of mutations involving positions 276 and 412 are easily explained by the fact that these are directly involved in metal binding. Positions 278 and 448 are in close proximity to these functional sites so it does not come as a surprise that they are critical.

**Figure 5:**
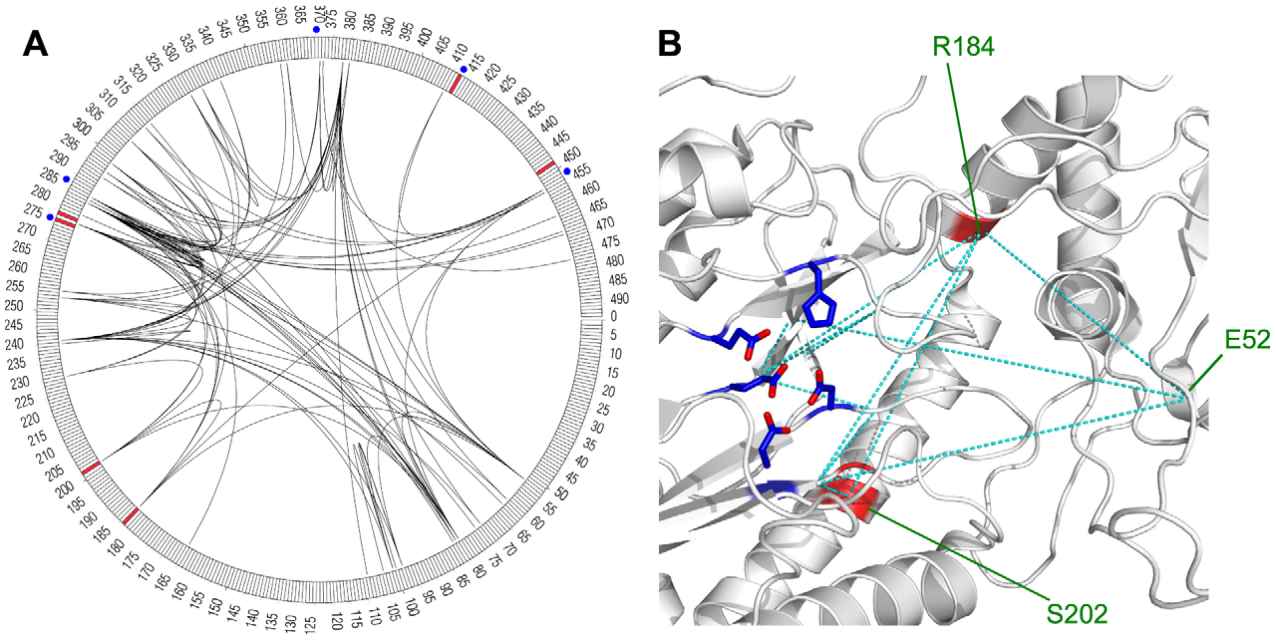
Correlation patterns in the human proline peptidase PEPD. (A) circular representation of linear protein sequence. Arcs indicate residue correlations. (B) Structure view indicating selected correlations as dashed lines. Metal binding residues are shown with side chains (PDB: 2iw2). (A,B) Disease associated positions are marked red, functional sites in blue.

R184 and S202 on the other hand, are situated far away from the metal binding sites in the primary sequence. While S202 gets close to the metal binding region in the three dimensional structure, R184 is located quite distant from this area. So why do point mutations at these sites cause disease? Of course, one possibility is that the enzyme function is destroyed by mechanisms totally unrelated to metal binding, but constructing a link to the important functional sites is another. We find that position 184 shows a strong co-evolution connection to positions 453 and 277 which are both in direct proximity of the metal binding residues (Figure 5a). Position 202 also shows a correlation with positions 277 and 184. We thus suggest that both R184Q and mutations of S202 do in fact inhibit manganese binding mediated by a non-contact interaction between these residues.

In summary, all known PD causing point mutations are either in close proximity to the critical residues or a correlated mutation link to such residues can be found. Figure 5b shows the spacial relations of the metal binding residues and some of the correlations found in this region.

Upon close inspection of the co-evolution connections depicted in Figure 5a, it is easy to see that the correlation links are not distributed evenly across the protein. Some residues or regions are connected to others by multiple arcs. Also, many positions are not only connected to each other but also share common neighbors hinting at a network of correlated positions. This observation seems to hold for the entire group of metal binding sites and disease positions discussed above: all of these positions seem to be part of a small correlation network.

### Adenylate kinase isoenzyme 1

Our second example is AK1, the human adenylate kinase isoenzyme 1. AK1 catalyzes the reversible transfer of the terminal phosphate group between ATP and AMP that is essential for cell maintenance and growth.

Point mutations of AK1 cause hemolytic anemia due to adenylate kinase deficiency (OMIM: 612631). AK1 catalyzes the reaction of ATP and AMP to two ADPs that is done by two nucleotide bindings regions. According to SwissProt annotation, the ATP binding site is located at residue 15–23 and the AMP binding site comprises residues 39 and 94–101.

**Figure 6:**
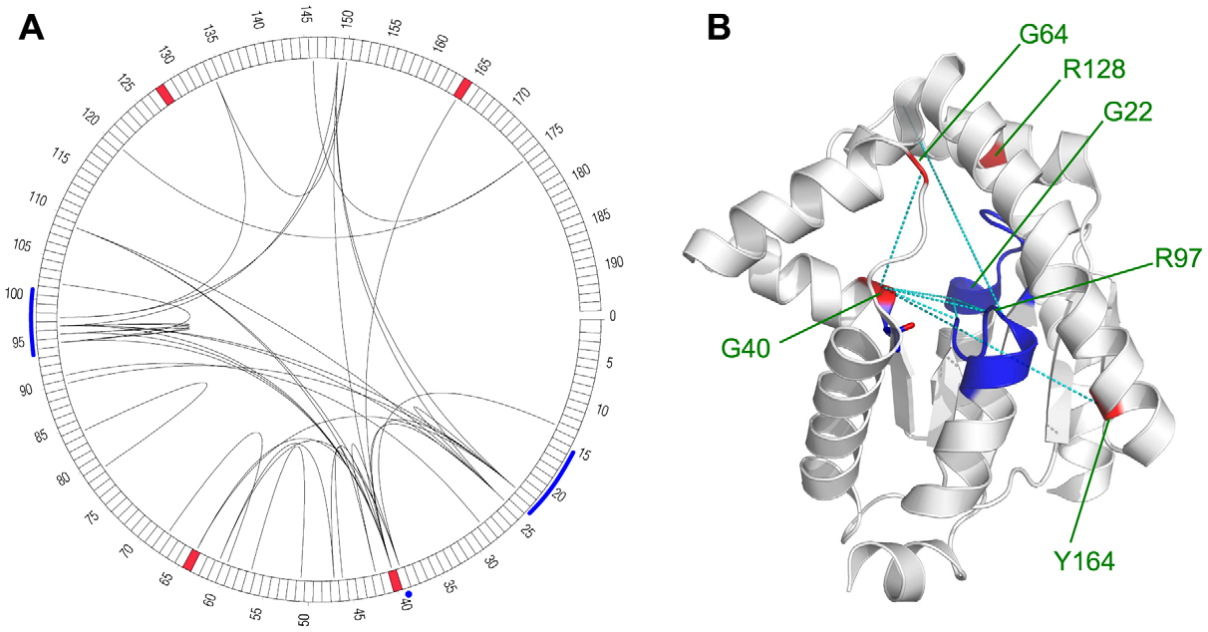
Correlation patterns in the adenylate kinase isoenzyme 1. (A) circular representation of linear protein sequence. Arcs indicate residue correlations. (B) Structure view indicating selected correlations as dashed lines (PDB: 2C95). (A,B) Disease associated positions are marked red, functional sites in blue.

Several different positions of the protein have been found to result in an altered phenotype upon mutation. Residue G40 is in direct neighbors of the nucleotide binding T39 while Y164 is somewhat further away. Some other positions (G64 and R128) are located in the neighborhood of these active sites in the 3D structure (Figure 6).

We find that residues implicated in disease phenotypes of AK1 are located at or near correlated positions which link to or close to the nucleotide bindings sites. As for the PEPD example, there appears to be a network of correlated positions. Residue G64 is directly linked to residue G40 that is a disease position itself and is immediately adjacent to one of the binding site T39. In addition, residue Y164 is directly linked to the disease residue G40. We found correlated links between both nucleotide binding regions and a direct link of G64 and G40 with residue G22 that is located in the ATP binding site. Obviously, disease relevant mutations can be “explained” by co-mutation links to binding sites or they are not involved in correlations themselves but are located in their close neighborhood (e.g. R128).

Both case studies illustrate that correlation networks may connect residues located elsewhere on the protein structure to a given disease associated mutation site. Disease affected positions and their immediate neighborhood tend to be connected in the network with other functionally important residues or regions such as metal binding sites or binding sites. In some other cases, disease-affected residues are located next to correlated residues or in well-connected regions of the network. These linkages could provide hints on the effects of disease-associated mutations as the corresponding networks could be used to transport the effects of point-mutations to functionally important regions. Accordingly, correlation networks provide a novel basis for selecting promising target residues for mutation studies or estimating the potential effects of yet uncharacterized naturally occurring mutations.

### Multiple correlation

**Figure 7:**
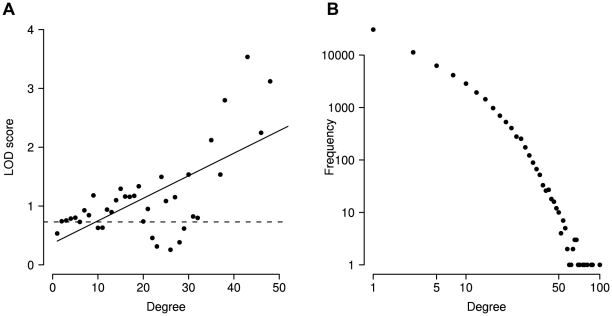
Multiple correlations. (A) The degree (number of correlation partners) of a position in the correlation network is positively correlated with the log odds of causing disease. As very large degrees are rare, substantial noise occurs at degrees above 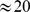. For lower values the association is clearly visible. Overall the association is substantial and significant (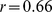, 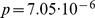). (B) Degree distribution of multiple correlations.

Above we mentioned small networks of correlated positions. From a graph perspective, positions with a higher degree (number of edges) should be more important than those with a low degree. In order to test if this also applies to our correlation network with respect to disease we analyzed the correlation graphs and found a clear association between the degree of a position and its probability to be associated with disease (Figure 7). Positions that are correlated with increasing numbers of other positions are increasingly more likely to be associated with disease (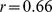, 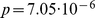).

## Discussion

In the past, analysis of residue co-evolution in proteins has been applied to various problems mainly centered around the idea of compensatory amino acid substitutions on protein contact surfaces. Correlations not readily explained by contacts have been discussed in the field and were simply labeled “noise” by many groups [27], [45], [46]. New ideas in this field have been introduced by many researchers and concepts such as a *correlation backbone* as an element of protein structure or mapping functional sites to correlation hotspots have been explored and illustrated by various examples. First discussions of disease relevance have only recently entered the literature and were restricted to selected proteins or protein families [18], [32]. To our knowledge, the data presented above represents the first attempt at a comprehensive analysis of evolutionary residue co-mutation in the light of disease associated point mutations. Our data indicate that residues highly correlated with others are indeed more likely to be associated with disease than expected. Surprisingly, as little as 30 orthologous sequences sufficed to detect a significant difference and considering only orthologous groups with at least 125 proteins did not yield a substantial difference.

Of course, a single parameter such as correlated evolution cannot be expected to yield a high positive predictive value when used in isolation but our findings clearly show that it is one property to look for when judging the functional significance and/or potential to cause a disease phenotype upon mutation. A fair assessment of the value of such a measure is probably the direct comparison to the most popular approach of using sequence conservation as an indicator of functional importance. As our analysis indicates, correlation seems to be associated with disease less than conservation but appears to be on the same order of magnitude.

This has clear practical implications. Some diseases are caused by large numbers of different point mutations in seemingly random locations in the protein. Analysis of co-evolution could serve as an interesting tool to explain some of these cases. Given the rapidly decreasing cost of sequencing, the hunt for SNPs and the trend towards personalized medicine, more and more data on variations of unknown physiological consequences will be gathered. Analysis of correlated mutations could prove to be a useful complement to data on conservation, structure and other protein features in the attempt to understand functional relevance of mutations.

We found that the correlated mutation detection was largely independent of conservation signal over a wide range and that the majority of correlations did not coincide with contacts in the subset of proteins for which protein structure data was available. Accordingly, we believe that the data indicate a genuine signal of co-evolution among functionally linked positions which are vulnerable to mutations. These findings are in line with the work on structural determination of protein domains based on the co-mutation signal [28] and provide good evidence for the concept of long-distance associations within proteins.

Another interesting observation which we have not yet analyzed systematically, is the role of “near-miss correlations”, i.e. correlations between residues in the direct neighborhood of functionally essential sites and/or known disease-associated positions. This concept is similar to our observation that sometimes point mutations causing disease are located next to critical residues without actually destroying them. In some cases one may argue that hitting the critical residue itself would be too drastic a defect to be viable, in others a simple mechanistic point of view arguing that local changes are likely to influence their direct neighborhood may suffice as an explanation.

Many conceptually different algorithms have been developed to detect residue co-mutation. Fodor et al. have shown that these algorithms have a preferred level of conservation to extract significantly correlated pairs [25]. In our work, we used the OMES algorithm, as it was found to be among the best performers and most robust methods for contact prediction. But that does not necessarily imply that OMES is the single best choice for the analysis of non-contact correlation or disease mutations under each and every condition. We have preliminary data which shows that other methods produce comparable LOD scores for the global analysis, but differ in their performance depending on the degree of conservation. In future work, we are planning to study this in more detail and take advantage of differential preferences.

Correlated behavior of contact residues is a plausible and well accepted concept but what mechanisms produce long-range correlations? Lapedes et al. have suggested subsequent pairs of contact residues which extend the co-variation along a chain of contact-correlations [47]. This model is in good agreement with the concept of networks of correlated positions like the ones seen in our case studies and other preliminary data.

Another possible explanation for long-range correlations is the impact on protein folds. It is conceivable that amino acid substitutions may change the orientation of neighboring secondary structure elements by a few degrees. Such a change could easily impair protein function. It is not too difficult to imagine another change on the other side of such our helix to compensate this structural change and thus keep the rest of the fold largely unaffected.

Russ et al. introduced the concept of correlated sites as an architectural backbone of protein domains [29]. Many of the correlations used in their model related to positions which are clearly not in contact with each other and thus forming a *correlation backbone*. This work suggest that co-evolving residues within a protein contain more information than the mere potential to be in physical contact.

Methods like OMES that solely operate on multiple sequence alignments in analyzing correlations have previously been criticized for being “tree agnostic” – i.e. ignoring the underlying phylogenetic tree in their assessment. Others have argued that no substantial gain is achieved by tree-aware methods [48]. In order to test if using the phylogenetic information substantially affects our results, we repeated all major analyses with the algorithm of Noivirt et al. [49]. Although this method yielded a slightly lower LOD than OMES (0.63 vs. 0.73), all of our conclusions could be confirmed in this re-analysis.

In summary, we believe that researchers should not only look at conservation in their judgment of functional significance of residues in the protein sequence. Correlation patterns between residues clearly provide additional evidence which should not be ignored.

## Materials and Methods

### Mutation data

Disease mutation data was obtained from the HGMD database [2], a comprehensive collection of mutations underlying human inherited disease. Access to the full HGMD release 7.3 was licensed from Biobase, Germany (http://www.biobase-international.com).

Out of a total of 73 411 mutation entries 41628 referred to point mutations, while the rest represented other types of mutation. From this initial set of 2253 human proteins, we eliminated all entries describing mutations leading to stop codons and thus truncation of the full length protein. The final data set comprised 32 923 disease-related amino acid substitutions affecting 27 522 unique positions in 2067 different proteins.

### Orthologous protein sets

Groups of orthologous proteins were downloaded from the STRING database (release 7.0) [50] where these were generated for their COG-interaction mode. Because some of these computationally derived orthologous groups contain a very broad range of sequence homology which in some cases may go beyond orthologs, we removed all sequences which failed to cover at least 80% of the human reference sequence in the multiple alignments according to STRING.

Often, such ortholog sets contain many sequences from very closely related species resulting in an excessive apparent sequence conservation and thus undue weight of almost identical sequences which does not reflect the evolutionary situation but the bias in protein selection. To overcome this bias, we iteratively removed near-identical sequences from the ortholog sets. We calculated the sequence identity of all pairs in global alignments. If two sequences were over 90% identical, one of them was picked at random.

Very small ortholog sets cannot reasonably be expected to allow valid conclusions about evolutionary correlation, because the resulting multiple alignments simply do not contain enough sequences. In the literature, a wide range of minimum ortholog clusters sizes have been applied. While some used as little as 15 proteins [21], [51], [52], other required more than 125 different orthologs [18], [53], [54]. In our study, we initially used all clusters with at least 30 orthologous proteins, which is a very common cutoff [53], [55]. In addition, we also performed our analysis on a more stringent data set by using a threshold of 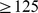. The former threshold yields 1153 human proteins with their orthologs, while the more strict cutoff still leaves us with 855 such clusters.

### Functional sites

We used the feature annotation from SWISSPROT [39], [40] to identify functionally relevant positions in each of the disease associated proteins with a sufficient number of orthologs. We included the following feature tags in our analysis: CA_BIND (calcium-binding), DNA_BIND (DNA binding), NP_BIND (nucleotide phosphate-binding), ACT_SITE (involved in enzyme activity), METAL (metal binding), BINDING (binding of unspecified chemical group), MOD_RES (posttranslational modification) and LIPID (lipid binding). In total, we obtained 12021 functional residues in 745 proteins.

### Multiple sequence alignments (MSA)

Alignments of orthologous proteins were carried out using MUSCLE 3.6 [56] with default parameters. To reduce computational requirements we limited each ortholog set to a maximum of 300 sequences, after filtering, by selecting a random sample of 299 sequences plus the human reference sequence, which always needs to be present for analysis.

### Correlated mutation analysis

**Figure 8:**
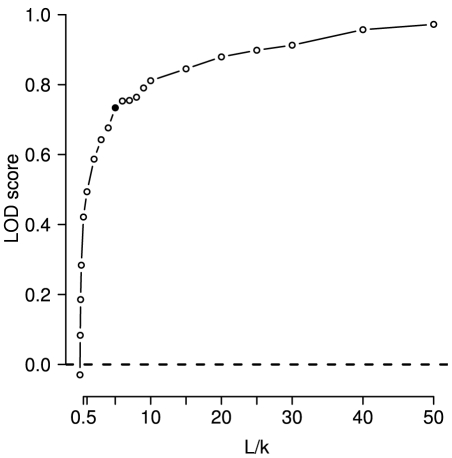
Influence of correlation cutoff 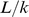. Commonly, 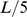 (i.e. 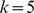) is used. At first a substantial increase of LOD can be observed with increasing values of 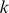. Once 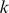 reaches values of 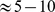 the steep increase is replaced by a quite moderate slope.

Correlated mutations were analyzed using the OMES (Observed Minus Expected Squared) algorithm. The OMES method is based on the 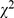 goodness-of-fit test and compares the observed co-occurrence of amino acid 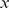 at position 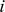 and amino acid 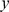 at position 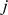 to the expected co-occurrence at positions 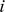 and 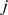 [20], [25], [57]. In this work we use the OMES variant defined by Fodor et al. [25]. We computed OMES correlation scores for all combinations of positions in each protein based on the multiple sequence alignments described above. Following the previously described approach we selected the top 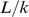 co-evolving residue pairs where 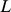 is the length of the respective protein and 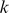 is a constant which is often set to 5 [55], [58]. To assess the influence of the constant 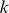 we evaluated our findings over a range of 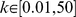. Based on this analysis, the commonly used value of 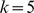 appears to be a good choice for our application: For values of 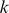 from 0 to 5 the observed LOD shows a steep increase. Somewhere around 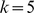 or 10 the curve adopts a much more moderate slope (Figure 8). A choice of 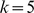 takes advantage of the initial LOD improvement without including excessive numbers of positions in each protein.

For further analysis, a sequence position was called *correlated* if it had at least one significant correlation with another position according to the above criteria.

### Sequence conservation

Two different measures of sequence conservation were computed for each position of a human protein. The BLOSUM conservation-score 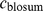 for each human residue was calculated by summing over the BLOSUM-scores for each residue pair between the human amino acid and all ortholog residues in the column of the MSA and normalizing to the maximum score for the given residue:
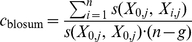
where 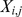 is the 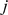
th residue of sequence 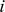 in the MSA; 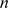 is the total number of sequences; 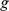 is the number of gaps in column 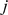; 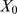 refers to the human reference sequence and 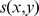 is the score for amino acids 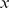 and 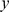 according to the BLOSUM62 scoring matrix [33]. As BLOSUM scores can be negative, 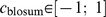


The second approach simply computes the fraction of residues identical to the reference sequence 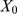 for column 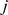 of the MSA:
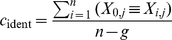



### Structural contacts

Protein structure analysis was performed for all proteins of the filtered set for which at least a partial crystal structure was available from the PDB database [59]. 238 proteins fulfilled this criterion. The spatial distance between correlated residue pairs was calculated taking into account all non-hydrogen side chain atoms of both amino acids. Two residues were considered to be in contact with each other, if the smallest distance between any pair of their non-hydrogen atoms was 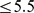 Å. This represents a commonly applied threshold – other groups have used distance cutoff in the range 5.0Å–8.0Å using the closest non-hydrogen or C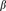 atoms, respectively [20], [53], [55]. In order to exclude local contacts in secondary structure elements, such as in 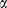-helices, we only considered residue pairs outside a window of 10 positions up- and downstream of a given position.

Some studies on correlated mutations have computed distances solely based on 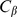 atoms (Glycine: 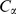) and a 8.0Å cutoff [25]. We provide additional data using this definition in the Text S1.

### Residue accessibility

The accessible surface area (ASA) was computed with DSSP [60] and converted to a relative solvent accessibility (RAS) by dividing by the maximum possible ASA of the respective amino acid. The data was discretized into three accessibility states: *buried* (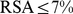); *intermediate* (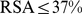) and *exposed* (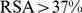).

### Identification of non-disease mutations

Amino acid substitutions with a low chance of causing harm were identified with a strategy used by [61]. The rationale of this approach is that amino acid substitutions tolerated during evolution are very unlikely to cause disease when observed in humans, at least for closely related species. We selected all human proteins from our set for which we were able to identify mammalian orthologs (from the ortholog clusters provided by the STRING database [50]) with at least 95% identity to the human sequence. Orthologs were only considered if they covered at least 80% of the human sequence in a pairwise alignment. 813 proteins satisfied both criteria. Amino acid substitutions found in the orthologs were considered non-damaging.

### Significance testing and bootstrapping

Statistical significance of enrichment of disease mutations in correlated positions was assessed by two different means. First we used Fisher's exact test for count data comparing the proportions of disease-annotated residues in correlated vs. non-correlated positions. In addition, we performed a 1000-fold permutation test in which we shuffled the disease/non-disease tags of the entire data set in order to obtain the empirical density function of the log odds (Figure 1). In order to assess the robustness of the observed LOD value we performed a 1000-fold bootstrapping of columns of the multiple sequence alignments. I.e. in each bootstrap we sampled, with replacement, from the columns of each of the multiple sequence alignments in the data set and then re-computed the LOD value for the entire re-sampled data set.

### Computation and visualization

All analysis programs for this work were written in Python, except for the program for correlated mutation analysis which was implemented in Java. Final data analysis and statistics was performed with the R statistical language [62]. Visualizations of correlations in the linear sequence were created with Circos [63]. Protein structure images were made with PyMOL (http://pymol.sourceforge.net/).

## Supporting Information

Text S1 Supplementary analysis.
(0.27 MB PDF)
