# Supplementary material for: Correlated Mutations: A Hallmark of Phenotypic Amino Acid Substitutions
Source: PLoS Comput Biol. 2010 Sep 16;6(9):e1000923. doi: 10.1371/journal.pcbi.1000923 (PMC2940720; doi:10.1371/journal.pcbi.1000923)
Supplement: Text S1 — Supplementary analysis. (0.27 MB PDF) [file pcbi.1000923.s001.pdf]

Supplement for "Correlated mutations: a hallmark of phenotypic amino acid substitutions"

Andreas Kowarsch, Angelika Fuchs, Dmitrij Frishman and Philipp Pagel

1 LOD score histograms for the data set with alignment threshold ≥ 125

1.1 Empirical background distribution

Empirical background distribution obtained by 1000 permutations. Vertical lines indicate the observed LOD obtained by data set using an alignment cutoff of ≥ 125 (dotted).

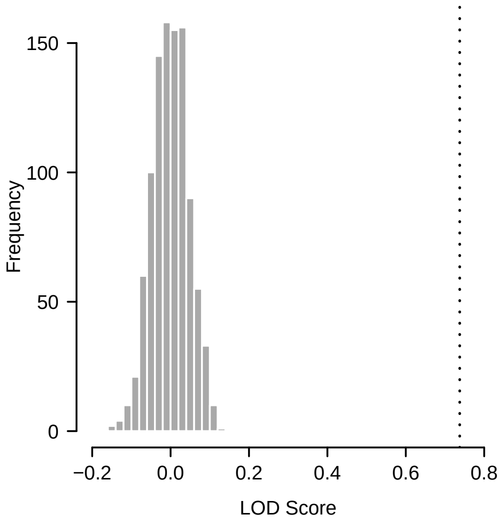

1.2 LOD distribution for individual proteins

1.2.1 All proteins

Distribution of log odds (LOD) scores for individual proteins using an alignment cutoff of ≥ 125 (equivalent to Figure 2a). All proteins were excluded for which no score could be obtained.

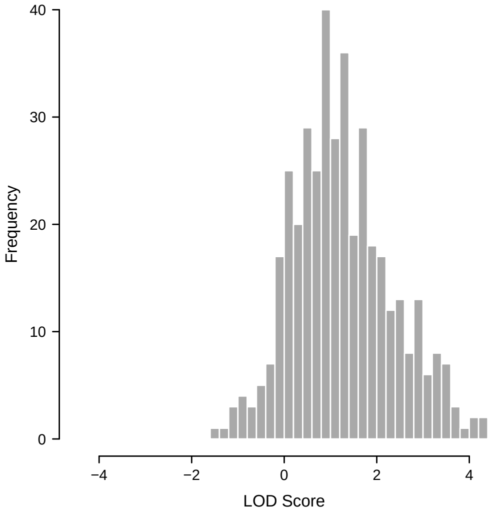

1.2.2 Proteins with ≥ 10 disease mutations

Distribution of log odds (LOD) scores for individual proteins using an alignment cutoff of ≥ 125 (equivalent to Figure 2b). LOD scores were plotted only for proteins with ≥ 10 known disease mutations in HGMD. All proteins were excluded for which no score could be obtained.

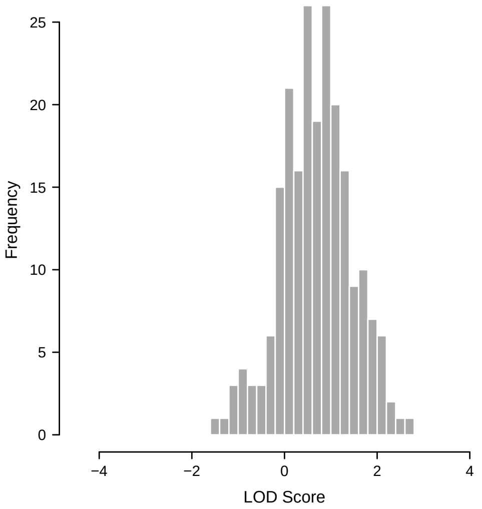

1.3 Interaction between correlation and conservation

1.3.1 BLOSSUM conservation score

Interaction between correlation and conservation. LOD distribution for conservation thresholds using the BLOSSUM conservation score (equivalent to Figure 3a). Each dot represents the LOD score achieved using a specific conservation cutoff. A cutoff of 0.4 indicates that for the calculation of the global LOD score only the residues which have a conservation score ≤ 0.4 were taken into account.

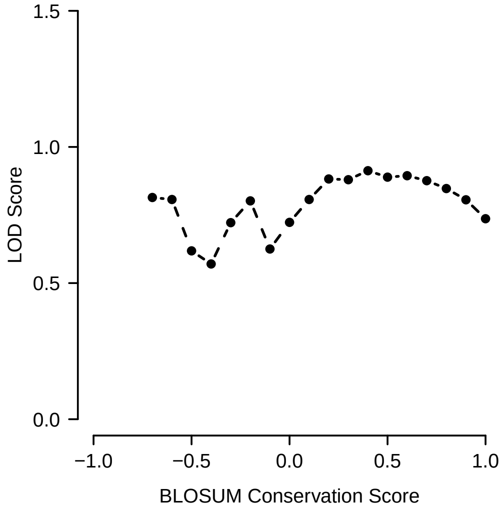

1.3.2 Fractional identity

Interaction between correlation and conservation. LOD distribution for conservation thresholds using the fractional identity (equivalent to Figure 3b). Each dot represents the LOD score achieved using a specific conservation cutoff. A cutoff of 0.4 indicates that for the calculation of the global LOD score only the residues which have a conservation score ≤ 0.4 were taken into account.

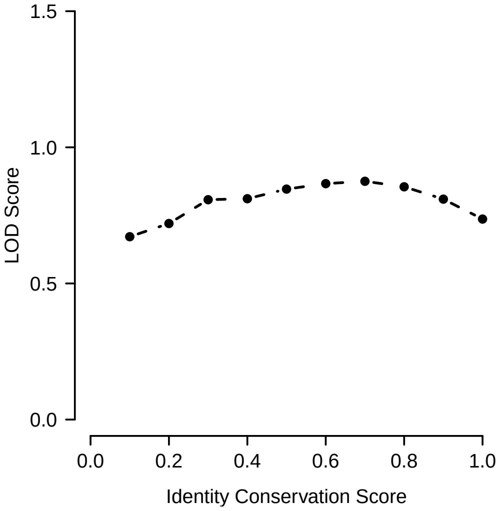

2 Summary table of spatial distance of residues

Analysis of spatial distance of residues. All: all residues; Disease: only disease affected residues.

subset	Distance-Threshold	Residues	Contact
All	$\leq$ 5.5	70589	59079 (83.7%)
Disease	$\leq$ 5.5	2747	2522 (91.8%)
All	$\leq$ 8.0	70589	59022 (83.6%)
Disease	$\leq$ 8.0	2747	2523 (91.8%)

3 Summary table of spatial distance of co-evolving positions

Distance analysis of co-evolving positions using different alignment cutoffs as well as distance thresholds. All: all significantly correlated residue pairs; Disease: significantly correlated residue pairs for which at least on residue is affected by a disease mutation; Corr-Pairs: Number of significantly correlated residue pairs; Contact: Number of significantly correlated contact pairs.

subset	Alignment Cutoff	Distance-Threshold	Corr-Pairs	Contact
All	30	$\leq$ 5.5	16555	3717 (16.4%)
Disease	30	$\leq$ 5.5	1636	261 (15.9%)
All	125	$\leq$ 5.5	13339	2342 (17.5%)
Disease	125	$\leq$ 5.5	1131	209(18.5%)
All	30	$\leq$ 8.0	16555	2887 (17.4%)
Disease	30	$\leq$ 8.0	1636	284 (17.4%)
All	125	$\leq$ 8.0	13339	2469 (18.5%)
Disease	125	$\leq$ 8.0	1131	219 (19.3%)

4 Summary of performance

4.1 Selection: fractional identity

Summary of performance for the fractional identity. P-values were computed using Fisher's exact test. T: number of all residues; C : number of positions; D: number of disease mutations; $D \wedge C$: number of disease-affected positions.

subset	T	D	C	D ∧ C	LOD	p-value
Conservation, (0.2)	741436	14211	351880	8726	0.37	$< 2.2 \cdot 10^{-16}$
Conservation, (0.5)	741436	14211	145829	4977	0.83	$< 2.2 \cdot 10^{-16}$
Conservation, (0.8)	741436	14211	51533	2357	1.25	$< 2.2 \cdot 10^{-16}$

4.2 Selection: contact residues

Summary of performance for contact residues. P-values were computed using Fisher's exact test. T: number of all residues; C: number of contact positions; D: number of disease mutations; $D \wedge C$: number of contact residues affected by disease-associated mutations.

subset	T	D	C	D ∧ C	LOD	p-value
Contact (≤ 5.5)	70589	2747	59079	2522	0.13	$< 2.2 \cdot 10^{-16}$
Contact (≤ 8.0)	70589	2747	59022	2523	0.13	$< 2.2 \cdot 10^{-16}$

4.3 Alignment cutoff ≥ 30, fractional identity

Summary of performance for the alignment cutoff ≥ 30 and using the fractional identity conservation score. P-values were computed using Fisher's exact test. T: number of all residues; C: number of correlated positions; D: number of disease mutations; $D \wedge C$: number of correlated residues affected by disease-associated mutations.

subset	T	D	C	D ∧ C	LOD	p-value
Correlation	741436	14211	62365	1988	0.73	$< 2.2 \cdot 10^{-16}$
Correlation, (0.2)	396202	5531	23787	567	0.77	$< 2.2 \cdot 10^{-16}$
Correlation, (0.5)	605072	9342	50105	1429	0.88	$< 2.2 \cdot 10^{-16}$
Correlation, (0.8)	691531	11869	62182	1980	0.89	$< 2.2 \cdot 10^{-16}$
Correlation, non-contact (> 5.5)	70589	2747	4960	252	0.38	0.0018
Correlation, non-contact (> 8.0)	70589	2747	5027	256	0.39	0.0016

4.4 Alignment cutoff ≥ 125, BLOSSUM conservation score

Summary of performance for the alignment cutoff ≥ 125 and using the BLOSSUM conservation score. P-values were computed using Fisher's exact test. T: number of all residues; C: number of correlated positions; D: number of disease mutations; $D \wedge C$: number of correlated residues affected by disease-associated mutations.

subset	T	D	C	D ∧ C	LOD	p-value
Correlation	538283	10508	46022	1498	0.73	$< 2.2 \cdot 10^{-16}$
Correlation, (0.0)	244185	3488	14121	335	0.72	$< 2.2 \cdot 10^{-16}$
Correlation, (0.5)	461193	7630	37573	1155	0.90	$< 2.2 \cdot 10^{-16}$
Correlation, (0.8)	512819	9187	45723	1478	0.85	$< 2.2 \cdot 10^{-16}$
Correlation, non-contact (> 5.5)	55156	1982	3922	166	0.23	0.14
Correlation, non-contact (> 8.0)	74459	2988	3969	167	0.23	0.16

4.5 Alignment cutoff ≥ 125, fractional identity

Summary of performance for the alignment cutoff ≥ 125 and using the fractional identity. P-values were computed using Fisher's exact test. T: number of all residues; C: number of correlated positions; D: number of disease mutations; $D \wedge C$: number of correlated residues affected by disease-associated mutations.

subset	T	D	C	D ∧ C	LOD	p-value
Correlation	538283	10508	46022	1498	0.73	$< 2.2 \cdot 10^{-16}$
Correlation, (0.2)	329385	4790	20527	494	0.72	$< 2.2 \cdot 10^{-16}$
Correlation, (0.5)	465174	7604	37743	1113	0.85	$< 2.2 \cdot 10^{-16}$
Correlation, (0.8)	513699	9215	45849	1492	0.85	$< 2.2 \cdot 10^{-16}$
Correlation, non-contact (> 5.5)	55156	1982	3922	166	0.23	0.14
Correlation, non-contact (> 8.0)	74459	2988	3969	167	0.23	0.16

5 LOD score histograms for functionally important residues (Swissprot annotation)

Functionally important residues based on Swissprot annotation. Following keyword were used: CA_BIND, DNA_BIND, NP_BIND, ACT_SITE, METAL, BINDING, MOD_RES, LIPID.

5.1 Empirical back-ground distribution

Empirical background distribution obtained by 1000 permutations. Vertical lines indicate the observed LOD.

5.1.1 Alignment cutoff ≥ 30

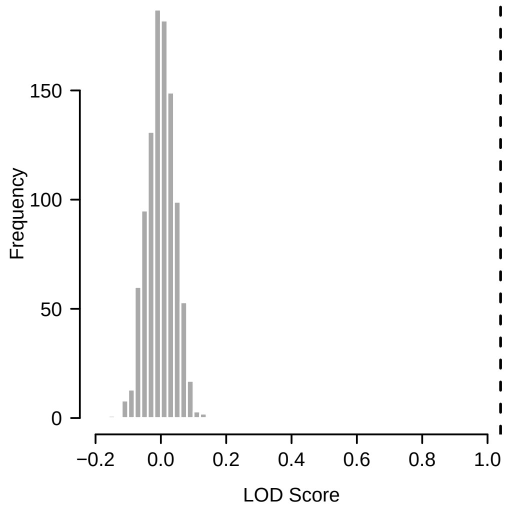

5.1.2 Alignment cutoff ≥ 125

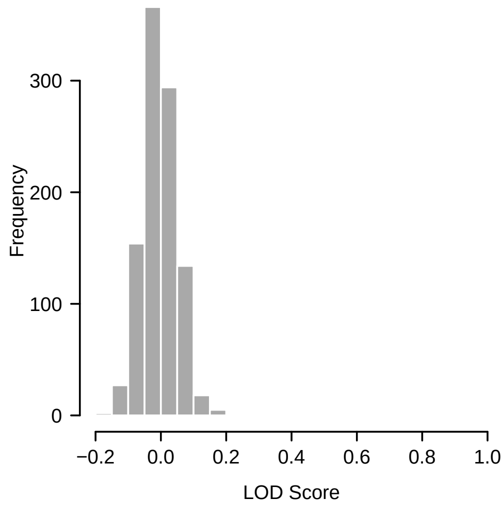

5.2 LOD distribution for individual proteins

Distribution of log odds (LOD) scores for individual proteins (equivalent to Figure 2a). All proteins were excluded for which no score could be obtained.

5.2.1 Alignment cutoff ≥ 30

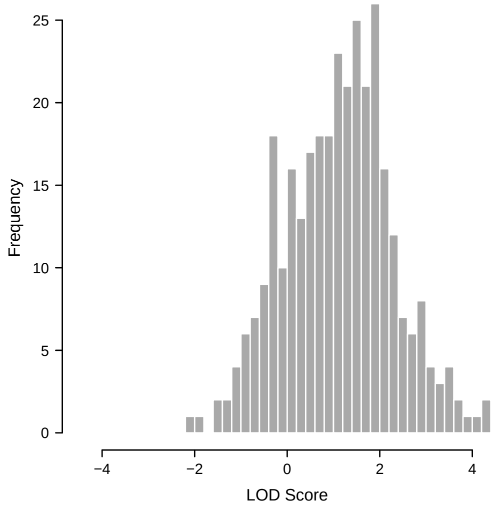

5.2.2 Alignment cutoff ≥ 125

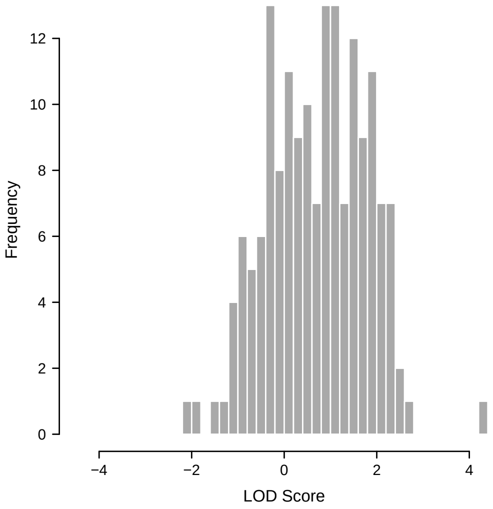

6 LOD score histograms for functionally important residues (Swissprot annotation + HGMD)

Functionally important residues based on Swissprot annotation and HGMD disease-associated point mutations.

6.1 Empirical background distribution

Empirical background distribution obtained by 1000 permutations. Vertical lines indicate the observed LOD.

6.1.1 Alignment cutoff ≥ 30

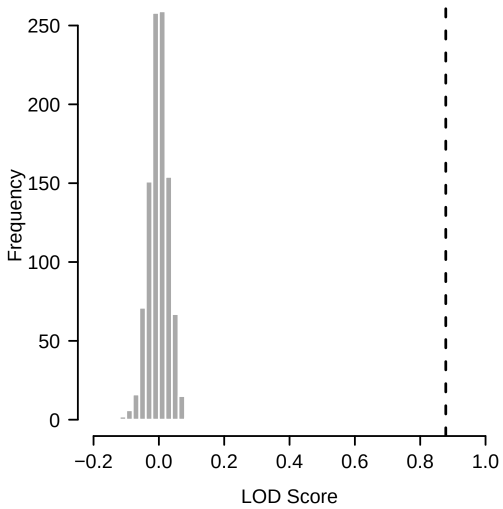

6.1.2 Alignment cutoff ≥ 125

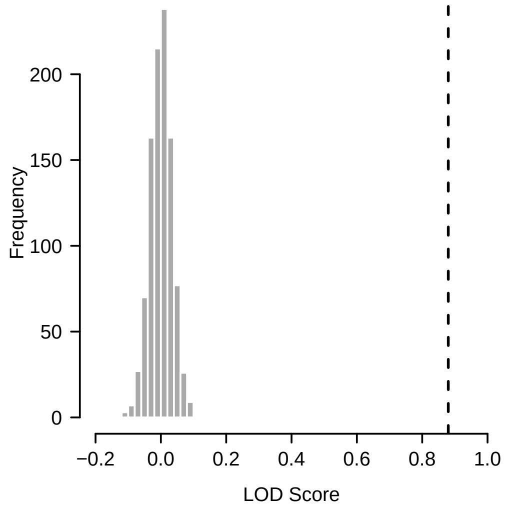

6.2 LOD distribution for individual proteins

Distribution of log odds (LOD) scores for individual proteins (equivalent to Figure 2a). All proteins were excluded for which no score could be obtained.

6.2.1 Alignment cutoff ≥ 30

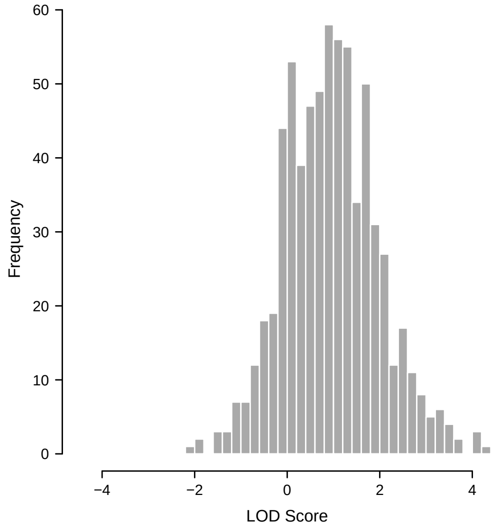

6.2.2 Alignment cutoff ≥ 125

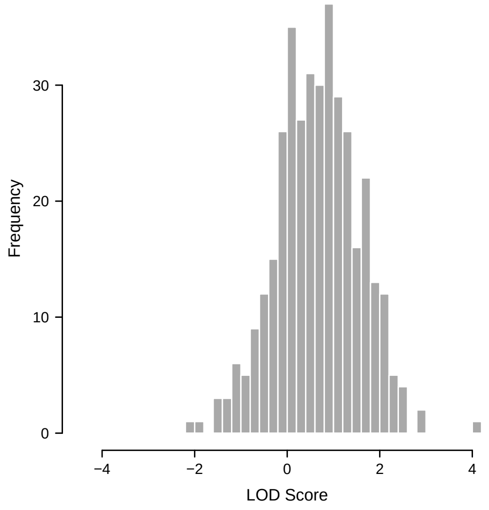

7 Results of Noivirt Method

In order to evaluate our results, we repeated our analysis with the method of Noivirt et al. Aside from slight differences in the specific numbers, this analysis fully confirms the conclusions obtained by the OMES method.

7.1 LOD distribution for individual proteins

7.1.1 All proteins

Distribution of log odds (LOD) scores for individual proteins using the Noivirt method (equivalent to Figure 2a). All proteins were excluded for which no score could be obtained.

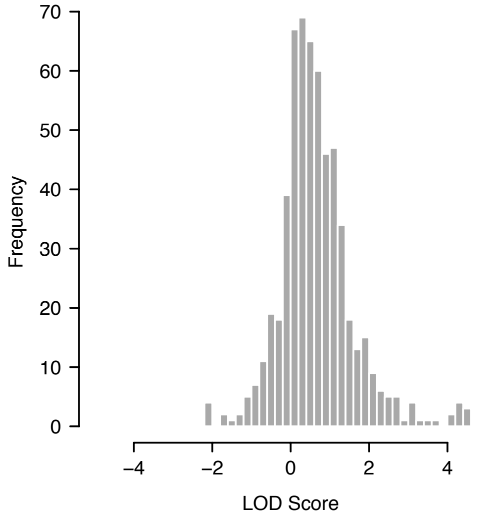

7.1.2 Proteins with $\geq$ 10 disease mutations

Distribution of log odds (LOD) scores for individual proteins using the Noivirt method (equivalent to Figure 2b). All proteins were excluded for which no score could be obtained. LOD scores were plotted only for proteins with $\geq$ 10 known disease mutations in HGMD. All proteins were excluded for which no score could be obtained.

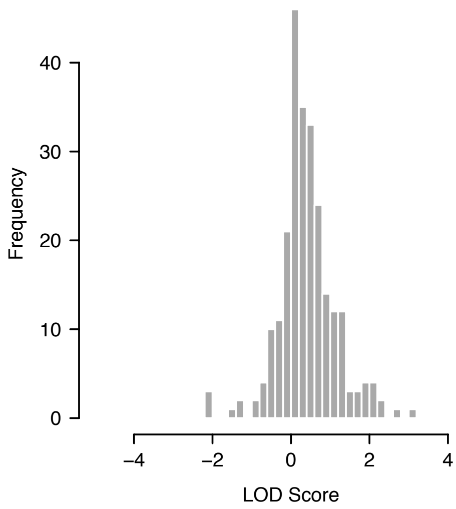

7.2 Interaction between correlation and conservation

7.2.1 BLOSSUM conservation score

Interaction between correlation and conservation. LOD distribution for conservation thresholds using the BLOSSUM conservation score (equivalent to Figure 3a). Each dot represents the LOD score achieved using a specific conservation cutoff. A cutoff of 0.4 indicates that for the calculation of the global LOD score only the residues which have a conservation score ≤ 0.4 were taken into account.

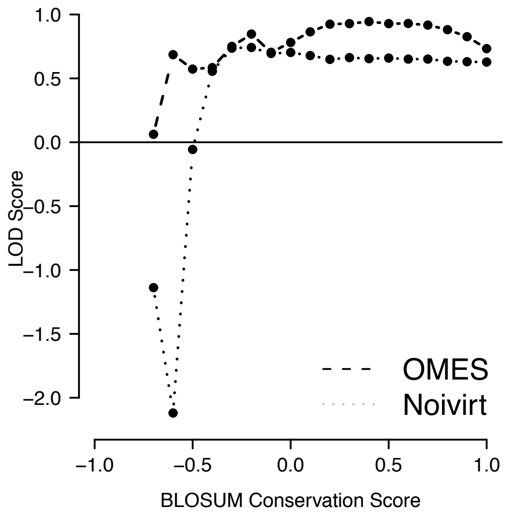

7.2.2 Fractional identity

Interaction between correlation and conservation. LOD distribution for conservation thresholds using the fractional identity (equivalent to Figure 3b). Each dot represents the LOD score achieved using a specific conservation cutoff. A cutoff of 0.4 indicates that for the calculation of the global LOD score only the residues which have a conservation score ≤ 0.4 were taken into account.

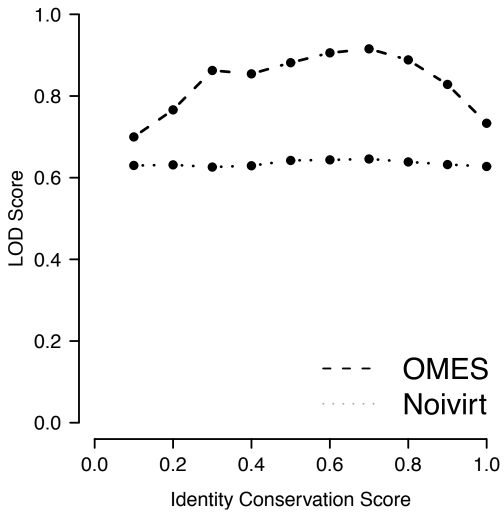

8 Results of McBASC

In order to evaluate our results, we repeated our analysis with the McBASC method (Göbel et al. 1994; Olmea and Valencia 1997). Aside from slight differences in the specific numbers, this analysis fully confirms the conclusions obtained by the OMES and Noivirt methods.

8.1 LOD distribution for individual proteins

8.1.1 All proteins

Distribution of log odds (LOD) scores for individual proteins using the Noivirt method (equivalent to Figure 2a). All proteins were excluded for which no score could be obtained.

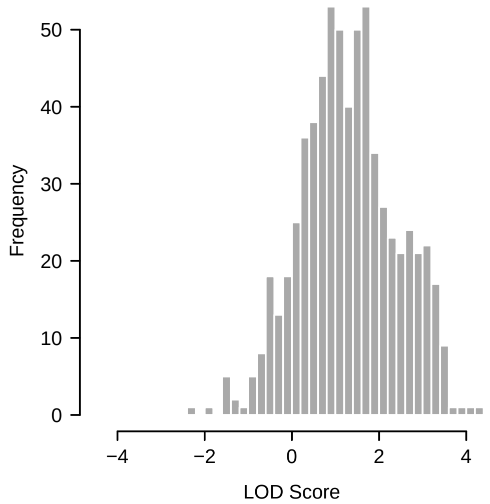

8.1.2 Proteins with $\geq$ 10 disease mutations

Distribution of log odds (LOD) scores for individual proteins using the Noivirt method (equivalent to Figure 2b). All proteins were excluded for which no score could be obtained. LOD scores were plotted only for proteins with $\geq$ 10 known disease mutations in HGMD. All proteins were excluded for which no score could be obtained.

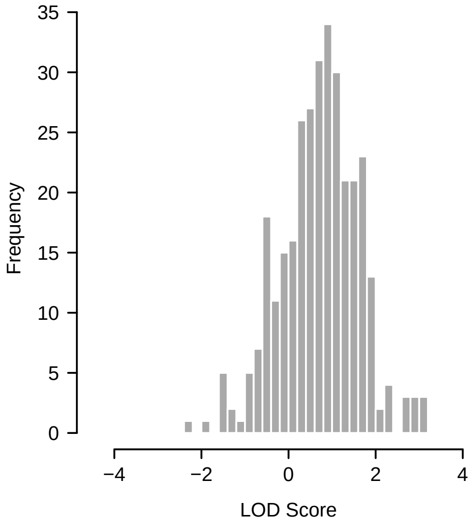

8.2 Interaction between correlation and conservation

8.2.1 BLOSSUM conservation score

Interaction between correlation and conservation. LOD distribution for conservation thresholds using the BLOSSUM conservation score (equivalent to Figure 3a). Each dot represents the LOD score achieved using a specific conservation cutoff. A cutoff of 0.4 indicates that for the calculation of the global LOD score only the residues which have a conservation score ≤ 0.4 were taken into account.

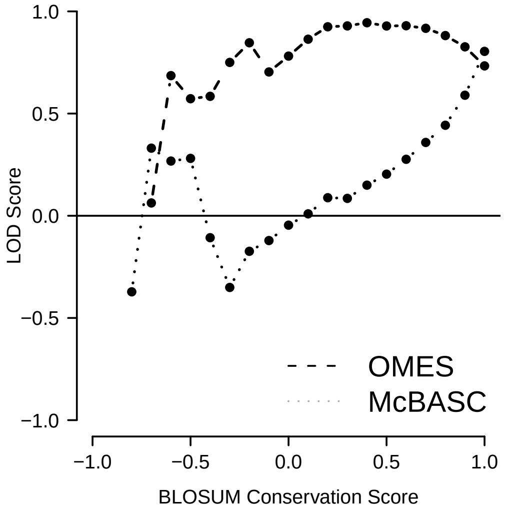

8.2.2 Fractional identity

Interaction between correlation and conservation. LOD distribution for conservation thresholds using the fractional identity (equivalent to Figure 3b). Each dot represents the LOD score achieved using a specific conservation cutoff. A cutoff of 0.4 indicates that for the calculation of the global LOD score only the residues which have a conservation score ≤ 0.4 were taken into account.

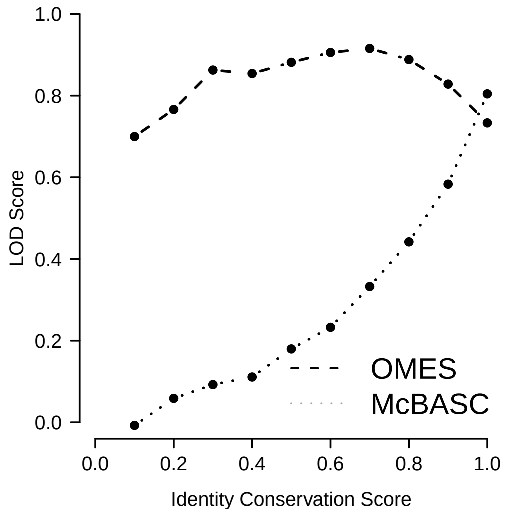

9 Comparison of different methods

Summary of performance for the OMES method using the alignment cutoff ≥ 30 and ≥ 125 as well as the Noivirt and McBASC methods. P-values were computed using Fisher's exact test. T: number of all residues; C: number of correlated positions; D: number of disease mutations; $D \wedge C$: number of correlated residues affected by disease-associated mutations.

Set	T	D	C	D $\wedge$ C	LOD	p-value
OMES ($\geq$ 30)	741436	14211	62365	1988	0.73	$< 2.2 \cdot 10^{-16}$
OMES ($\geq$ 125)	538283	10508	46022	1498	0.73	$< 2.2 \cdot 10^{-16}$
Noivirt	699882	13671	154840	4675	0.63	$< 2.2 \cdot 10^{-16}$
McBASC	935439	16085	99391	3001	0.81	$< 2.2 \cdot 10^{-16}$

10 Protein interfaces

Protein-protein interactions and therefore protein interfaces play a central role in health and disease. In order to validate whether correlated mutation located within an interface are enriched disease-associated we analyze the set of human disease proteins for which at least a partial crystal structure was available from the PDB database. We used the meta-PPISP meta-server [Qin and Zhou, 2007] which is based on three individual web servers: cons-PPISP, PINUP, and Promate to predict protein interfaces. Using the meta-server, we finally obtained a predictions for 191 human disease proteins.

Using all residues represented in the datasets described above, we produced contingency tables of correlatedness vs. known disease-mutations. Based on these tables, we computed the background rates of disease mutations to be 0.038% for random positions and 0.048% for correlated positions. In other words, we find known disease positions weakly enriched in protein interfaces than expected by chance (LOD = 0.33, $p = 7.26 \cdot 10^{-4}$).

11 Bootstrapping approach

In order to evaluate the error around the LOD values we conducted three slightly different bootstrapping experiments involving 1000 samples (with replacement), each.

11.1

In the first experiment, bootstrap samples were generated separately for each protein: I.e. positions (columns in the multiple sequence alignments) were randomy sampled separately for each protein (i.e.

MSA). Then the LOD values were computed for each bootsraped data set.

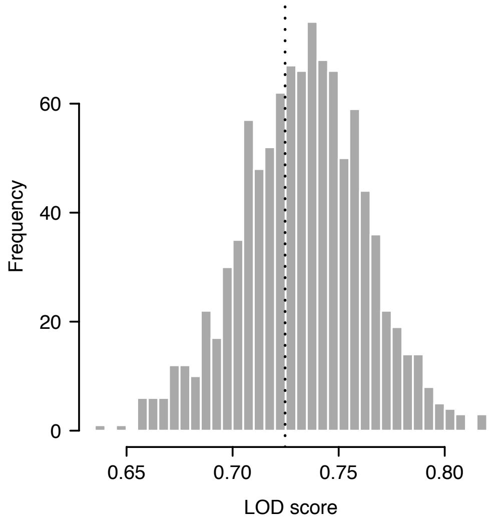

11.2

In the second experiment, entire MSAs were sampled. I.e. we randomly chose entire proteins (and not positions/MSA columns) from the original dataset without changing the composition of the individual MSAs.

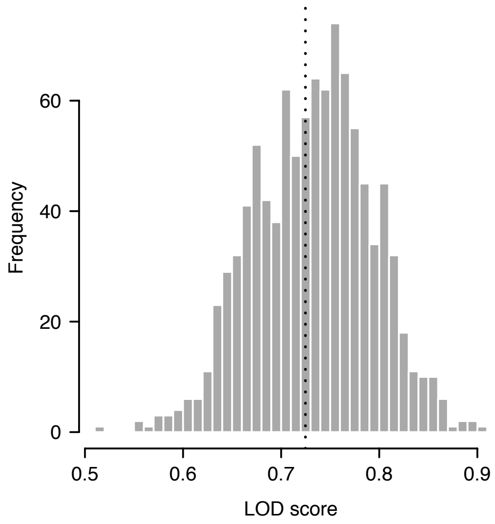

11.3

In the third experiment, we first fused all MSA alignments into one giant MSA containing all protein sequences and then proceeded sampling random columns from the concatenated MSA. This approach is different from the first one, because the whole dataset is treated like single giant sequence instead of obeying the protein boundaries.

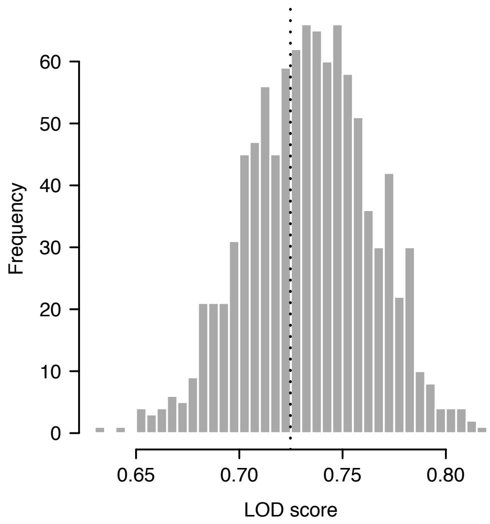

For each of these new data sets, the corresponding LOD score is calculated. The vertical line indicates the obtained LOD score for the OMES method using the alignment cutoff ≥ 30.
